# What do parents of nonverbal and minimally verbal autistic children think about genomic autism research?

**DOI:** 10.1177/13623613231213431

**Published:** 2024-03-09

**Authors:** Kathryn Asbury, Umar Toseeb, Naomi Barrow

**Affiliations:** 1 University of York, UK; 2 Independent Researcher, UK

**Keywords:** autism, genomics, Genome-Wide Association Studies, inclusion, minimally verbal, nonverbal, qualitative, Spectrum 10 K

## Abstract

Concerns have been raised about genomic studies of autism. Most recently, the Spectrum 10 K study was paused due to criticism from the autistic community. This situation raised important questions about how the autistic and autism communities perceive genomic research. The Personal Experiences of Autism and Perceptions of DNA-based-research study was established to address this issue. Twenty parents of nonverbal or minimally verbal autistic children took part in the current study. Data were provided in diverse formats including online interviews, telephone interviews, and writing. This approach was co-produced with autistic experts by experience. Data were analysed using reflexive Thematic Analysis. We found that participants were supportive of autism research, including some genomic research, if it is designed to support autistic people and is ethical and transparent. However, while some believed that polygenic scores, genomic predictors of the statistical probability of being autistic, would be helpful, others argued that this would only be true in an ideal world. Participants felt that they and their children were often excluded from, and unrepresented by, the autistic and autism communities. We conclude that genomic researchers need to work with the autistic and autism communities to design future work, and that it is important to ensure a representative range of voices are heard.

**Lay abstract:**

In Summer 2021, a genomic study of autism, Spectrum 10 K, was paused due to backlash from the autistic and autism communities. This raised important questions about how these communities perceive genomic research. The Personal Experiences of Autism and Perceptions of DNA-based research study was established to address this issue among a range of sub-groups within these communities. Twenty parents of nonverbal or minimally verbal autistic children took part in the current study. Data were provided in diverse formats including online interviews, telephone interviews, and writing. This approach was co-produced with autistic experts by experience and involved a parent of a minimally verbal autistic child. Data were analysed using reflexive Thematic Analysis. We found that participants were supportive of autism research, including some genomic research, as long as it is designed to support autistic people and is ethical and transparent. However, while some believed that polygenic scores, genomic predictors of the statistical probability of being autistic, would be helpful, others argued that this would only be true in an ideal world and that the world is too far from ideal. Participants felt excluded from the autistic and autism communities and that the dominant voices in those communities do not represent them or their children. We concluded that genomic researchers need to work with the autistic and autism communities to design future work, and that it is important to ensure a representative range of voices are heard.

Geneticists have been fascinated by autism since early twin studies found it to be a highly heritable condition (Folstein & Rutter, 1977), a finding that has been replicated many times since (Ronald & Hoekstra, 2011; Sandin et al., 2017; Tick et al., 2016). In recent years, twin studies have given way to Genome-Wide Association Studies (GWAS), with several major genomic autism studies launched in the last decade. GWAS rely on large samples to identify genetic variants that have individually minuscule effects but which, when combined in polygenic scores, can provide meaningful prediction of individual differences (e.g., Okbay et al., 2022) and might potentially, in the future, be used to estimate the statistical probability of infants being autistic.

Although autism research appears to be highly valued by autistic people, there are ongoing debates about the types of research that are perceived as valuable. For example, there have been recent calls for a focus on more applied research, with an emphasis on care and support and less of a focus on basic science (Frankish & Horton, 2022). Within that context, few strands of research have proved as controversial as genomic studies of autism, and this is particularly well exemplified by the Spectrum 10 K study. Launched in August 2021 with £3.2 million funding from the UK’s Wellcome Trust, Spectrum 10 K’s stated aim is to ‘investigate the genetic and environmental factors that contribute to autism and related physical and mental health conditions to better understand wellbeing in autistic people and their families’ (https://spectrum10k.org/about-spectrum-10k/). Within 1 month of its launch, the Spectrum 10 K team had been forced to pause for a period of consultation. This was a direct result of a substantial and sustained backlash from the autistic and broader autism communities, spearheaded by the grassroots Boycott 10 K campaign. At the time of writing, the consultation is still underway and the project has not been resumed.

Those who protested against Spectrum 10 K expressed concerns about the ethics and communication of the study but also feared that it represented a eugenic approach, rooted in the medical model, that could prove to be a backdoor to prenatal screening and the eradication of autism (Natri, 2021). In this sense, it was seen as a genuinely existential threat. Given the enormity of this prospect, it is not surprising that some representatives of the autistic and autism communities became highly vocal in their protests against Spectrum 10 K. These protests were clearly successful, leading to the Spectrum 10 K project being paused indefinitely. What is less clear is how representative of the autistic and autism communities these vocal representatives were.

This raises an important question about the extent to which the ‘heard’ voices of autism activists and those with a strong online presence represent the voices of other ‘less heard’ members of the autistic and autism communities. It leads one to ask whether the majority of members of these communities feel the same way about genomic studies of autism and, if not, whether there are differences between sub-groups. This led our team to develop the Personal Experiences of Autism and Perceptions of DNA-based-research (PEAPOD) study. The PEAPOD study conducts qualitative research with groups of individuals in the highly heterogeneous autistic and autism communities. The focus of the current article is parents of nonverbal or minimally verbal autistic children. We chose to study parents of nonverbal and minimally verbal autistic children on the basis that their experiences of autism might be different from those of parents of autistic children who are verbal and other groups of autistic children and adults.

Parents of autistic children have been found to be generally supportive of autism research (e.g., Chen et al., 2013). There have been studies of what parents of autistic children think about diagnostic genetic testing for autism, but much less focus on what they think about the possibility of DNA-based predictive screening.

Parents have been largely positive about the idea of diagnostic genetic testing for autism. Perceived benefits include an enhanced possibility of early intervention, improved aetiological understanding, more informed family planning, and the opportunity to support research (Chen et al., 2013). For example, Wagner et al. (2020) found that 96% of their sample of US parents were interested in DNA testing for their children. Similarly, Johannessen et al. (2017), in their Norwegian sample, found that parents were largely positive about the benefits of diagnostic genetic testing and viewed understanding more about the aetiology of autism as the primary benefit of it. However, a later study by the same group (Johannessen et al., 2022) identified that just over 50% of parents were also concerned about the ‘right not to know’ (Andorno, 2004).

It is interesting to note some evidence which suggests that the perceived benefits of diagnostic genetic testing may change depending on whether children have actually experienced genetic testing. Lucas et al. (2022) studied a group of parents who were waiting for genetic testing for their children and a second group who had received the results of a genetic test. While both groups were positive about the benefits of diagnostic genetic testing, those who had not yet experienced it were more likely to cite improved healthcare and increased access to therapies as expected benefits, whereas those who had received test results tended to see the primary benefits as being a better understanding of their child’s diagnosis and an improved understanding of its aetiology. This is relevant to our understanding of what individuals expect the outcomes of a test to be and whether those expectations are realistic.

While research suggests that parents of autistic children are broadly positive about research and diagnostic genetic testing for autism, there are signs that they are much less positive about the idea of prenatal genetic testing (Johannessen et al., 2017). GWAS offers something very different to either of these options, namely postnatal testing for the purposes of screening for the probability of children being autistic. To our knowledge, no study to date has looked at parents’ perceptions of this possibility, which has been brought about by the rise of GWAS.

This is an important question to ask now because GWAS are progressing very rapidly. GWAS of traits such as educational attainment (Okbay et al., 2022) suggest that once sufficiently powerful sample sizes are in place, it is reasonable to predict that polygenic scores for the probability of children being autistic could be developed. Such scores represent potential risks as well as potential benefits, and it is important that the autistic and autism communities are heard in discussions about the potential usefulness of such an approach.

Therefore, in the current study, we ask: How do parents feel about their nonverbal or minimally verbal autistic children participating in genomic studies of autism and, relatedly, how do they feel about the development of polygenic scores to predict autism?

## Methods

### Participants, recruitment, and data collection

Participants were 20 parents of nonverbal or minimally verbal autistic children (aged 4–11) attending primary schools in England (See Table 1). They were parents to 7 girls and 14 boys (1 participant had 2 autistic children who met the criteria). Children represented all primary school year groups from Reception (age 4–5) through to Year 6 (age 10–11), with at least two children in each year group. Although we did not ask about the type of school these children attended, it became clear in the interviews that the children of most participants attended either a special school or a specialist unit attached to a mainstream school (15/20 with one child in a mainstream school, one planning to move from mainstream to special and three cases where the information was unclear). The vast majority of participants were mothers (19/20). Of these, three identified as autistic, eight as neurotypical with suspected neurodivergence, and seven as neurotypical. Two participants did not answer this question.

**Table 1.** Sample characteristics.

Characteristic	
Parental role	19 mothers, 1 father
Child gender^a^	14 male, 7 female
School type	15 specialist provision, 2 mainstream, 3 undisclosed
Child school year^b^	Reception (4); Y1 (3); Y2 (2); Y3 (3); Y4 (4); Y5 (3); Y6 (2)
Data collection method – written	3
Data collection method – online video	8
Data collection method – phone	8
Data collection method – audio recording	1

a Our 20 parent participants were discussing 21 children as one had two autistic children who met our criteria.

b UK primary school year groups range from Reception (in which children turn 4 years old) to Year 6 (in which children turn 11 years old).

Recruitment was conducted via social media, with adverts shared on Twitter, by email to schools and autism groups, and in relevant Facebook groups. Interested parents were asked to complete a short online questionnaire to confirm the name and age of their child and that their child was autistic and nonverbal/minimally verbal. This generated 72 eligible expressions of interest, including a few that made contact via email rather than via Qualtrics. We sorted the replies on the basis of the child’s school year group, in an attempt to gather data from parents of children at all stages of primary school. We then selected participants semi-randomly within those smaller groups, that is, we chose every second or third participant (depending on group size) but also deviated from this slightly at times to ensure that we included a parent of a girl in as many year groups as possible and that we were gathering data from participants who had chosen a range of data collection types (see below). Our target sample was 18–22 participants, and we contacted people who had expressed interest, and were eligible, until we had 20 who provided data. We gave participants the opportunity to choose whether they would prefer to provide data through an online video call (camera off or on), via a telephone call or face-to-face. They were also offered the option of being interviewed by a friend or family member using our questions, providing written answers to a series of written questions or audio-record themselves answering written questions (see our OSF project page for the data collection tools, that is, interview schedule and equivalent schedule for written and audio-recorded responses https://osf.io/fg6mt/?view_only=66db7ccc644143c0af5b66f74137af87). In our final sample (*n* = 20), three participants chose to provide written data, one to provide audio-recorded answers to the questions, eight to provide data via a recorded Zoom video call (camera on) and eight to provide data via a recorded Zoom phone call. All interviews were conducted by the same researcher, and all participants received a shopping voucher in thanks for their time.

Written responses were anonymised by the research team, and recorded responses were transcribed and anonymised by an external transcription company.

### Coding and analysis

Written responses and interview transcripts were coded inductively and analysed using reflexive Thematic Analysis (Clarke & Braun, 2021). This involved an initial process of immersion in the data, taking field notes and discussing early thoughts with members of the PEAPOD team. This was followed by inductive coding of the full dataset, generating 55 codes, and curation of the data extracts that support them. This summary of codes was then reviewed and revised, with some codes being combined or deleted to avoid redundancy, leaving a total of 45 codes. The full dataset was then revisited for a second round of coding. At this point, only minor tweaks were being made to the codes so we proceeded to generate preliminary themes. After reviewing these provisional themes in relation to the data, we made minor revisions and developed clear descriptions of each theme.

### Community involvement statement

This project was developed by members of the PEAPOD team, including two adult autistic experts by experience and one researcher who is a parent of a minimally verbal autistic child. One of the experts, by experience, was closely involved in applying for ethical approval, helping to develop informed consent sheets and to design our inclusive approach to data collection (i.e., offering multiple methods of being involved). Both experts, by experience, were involved in co-producing interview questions and discussing data and findings in follow-up meetings, and both were paid for their time. The parent of a minimally verbal autistic child conducted the interviews and sometimes disclosed this shared experience to participants when it felt appropriate or helpful, for example, when a participant expressed concern about being judged for a reaction they described.

## Results and discussion

We developed five themes which describe how parents of nonverbal or minimally verbal autistic children perceive genomic autism research and how they feel about the development and use of polygenic scores for autism.

### We love autism research, but not necessarily all of it

Many participants expressed a high degree of openness to autism research and were willing for their children to participate in genomic studies of autism. However, their enthusiasm was not without limits, and participants were keen to understand the specific aims of individual studies.

#### Positive about genomic research

It was interesting to note the extent to which participants were interested in basic scientific research as well as applied research into autism. P9 said: ‘I just think the more information you can find out, the better. I don’t think there are any downsides’. This enthusiasm was driven by interest, curiosity, and desire to know more – and indeed for more to be known – about a phenomenon that had a major impact on their families. This is an interesting point to reflect on in relation to a recent paper about the types of autism research we should prioritise (Frankish & Horton, 2022).

Some participants acknowledged that they did not know a great deal about genomic research and were keen to learn more, potentially by participating in a study. Some of these felt unable to express a confident view on whether they saw the development of polygenic scores to predict the likelihood of autism as a good thing or a bad thing, highlighting the importance of effective communication and education in this area. ‘I mean I’m a bit on the fence with it. It all depends on what is the outcome and how the outcome is achieved, but I’m open to it; I’m not closed’ (P10).

Overall, we observed support for basic science genomic research into autism, but with significant caveats.

#### What we want, and what we don’t

The most notable concern participants expressed was that genomic studies of autism should not work towards eradication or cure. Instead, they were clear that research should be designed to enhance knowledge and understanding or to enhance and optimise the support available to autistic children and adults. ‘Yes, it all comes down to what the intended outcome is, really, and how they see that that would benefit the autistic community, rather than benefit the non-autistic rest of the world that might see it as just a problem’ (P16).

Two specific areas in which participants said that genomic studies would be welcome were sensory challenges and being nonverbal. This perspective offers an interesting insight into the perceived benefits of breaking the broad autism diagnosis down into a series of specific manifestations. It also sheds light on some of the experiences or aspects of behaviour for which individuals, and where relevant, their caregivers, may feel they need the most support. One participant who expressed a general distrust of genomic research said they may be open to a well-justified DNA-based study of sensory behaviour:

if they were looking for say when we look at sensory needs of people with autism, if they were trying to map that onto some DNA profile so that you could do better early diagnosis and to provide sensory supports . . . I’d go along with that one because it’s about supporting people and it’s about early identification which are both really important (P8).

Several other participants were keen to see genomic research into why some autistic children do not speak, or speak very little:

for us as a family, the non-verbal is the biggest challenge we have. And, although it kind of runs side by side, we almost see it as separate to the autism. So, you know the stims, and the flapping, and the sounds, and the lining up of things, and everything has to have an order, and stuff, we deal with that, we enjoy and embrace that. That’s fine for us, it makes no difference to any of us really, but the non-verbal is really the biggest challenge (P16).

Overall, participants were enthusiastic about research designed to support autistic children and adults, or simply to understand their autism, but not research designed to ‘fix’ them. A substantial proportion of participants were willing for their child to take part in a genomic study of autism.

### We care about the means as well as the ends

This theme is about the elements of research design and ethics that are particularly important to these participants.

#### Logistical concerns

The most commonly expressed concern about participating in a genomic study was that the children would have to provide a blood sample. This was based on traumatic experiences that had led to participants being unwilling to put their children through that level of distress again, unless it was absolutely necessary. ‘He hasn’t had bloods for a long time. When we last did . . . it did take a good few of us to kind of pin him down. It was just quite traumatic’ (P11). Once the interviewer reassured participants that DNA could be extracted without a blood test, they became much more relaxed. Given that a blood test was a dealbreaker for many of these participants, it is important for researchers to be very explicit from the outset that this is not necessary.

A second logistical concern was time. These are families who have a lot of appointments and a lot of child-related calls on their time. ‘I would like to know how long the study would take, whether we could mail the DNA sample to you . . ’ (P7). Again, it is important for researchers to make clear that participation can happen from home at a time that is convenient to participants.

In discussing participation in genomic research, some participants explained that their child had already experienced genetic testing to help with diagnosis, usually via a blood test. In most cases, they said that the genetic testing did not lead to clear answers and certainly not to additional support. However, this did not seem to put them off taking part in genomic research, as was also the case in Lucas et al. (2022).

#### Research ethics and transparent communication are paramount

Several participants expressed concerns about who would be doing the research, and who would be funding it, and said they would only want to be involved if they trusted the researchers and their source of income. ‘Yeah, I would look at what the research was connected with. I’d want to look into the organisation and any other research done by that centre and what their philosophy was’ (P8). This chimes with the reaction to Spectrum 10 K in which we know that distrust of particular research teams was a factor (Natri, 2021).

Some also said that, if they did take part, they would only want their data to be used in that particular study:

I still don’t know whether I would consent to any studies, to be honest, but if they got to a point where they could give me that level of reassurance it would be, I would only consent for that study and nothing else in the future (P17).

This was an issue that was also raised by activists against Spectrum 10 K, and it is an important one for genomic researchers to grapple with. In this era of big data and open science it has become commonplace to share data with multiple research teams, often asking quite different questions to those asked by the original study. In spite of the inconvenience involved, it seems important to give participants the choice of having their DNA destroyed after the study, keeping it but seeking consent to use it in any subsequent study; or to opt in to all future studies. This is perhaps even more understandable in the case of parents who are providing consent on behalf of children who may not be able to do so themselves.

A further request was that full information should be provided regarding the specific aims of the research, with a perception expressed that there is a lack of openness and transparency in this area. ‘I don’t know what they’re trying to achieve by having that DNA. There’s too many questions . . . I think . . . and not a lot of openness and transparency of what they want to use it for’ (P17).

Overall, this theme adds to Theme 1 in making a case that participants were often open to their child being involved in a genomic study of autism as long as their child’s needs were met and their own concerns addressed.

### Predicting likelihood of being autistic from birth would make life better

A good proportion of participants expressed a belief that polygenic prediction of autism at birth could have made life easier for them and their children. They felt this way even though the interviewer explained that a polygenic score could only ever predict probabilities and would not serve as a diagnostic tool.

#### Knowledge is power

The strongest and most widespread belief expressed in this sub-theme was that polygenic prediction at birth would help families to prepare for the experience of parenting their child. Participants said that if they had known there was an increased likelihood of their child being autistic, they would have taken the time to educate themselves and to prepare for a future that turned out to be very different to the one they had imagined. ‘What if I had had that test and known there was a higher chance? Then I might have been able to spot it sooner and given my child support sooner’ (P15).

Participants felt this preparation time would have benefitted their children. ‘So, it wouldn’t have changed her outcome but it may have made us less stressed and more patient, and that in itself can only have had a positive impact on her can’t it?’ (P6). They also believed that having this possibility in the back of their minds would have helped to reduce the guilt, anxiety, and uncertainty they experienced when their child was not developing in line with neurotypical milestones:

We wouldn’t have had to go through three years or four years of, ‘What is happening? Is there something really wrong with him? Did I do something that wasn’t right? Did I eat something that wasn’t right?’ We wouldn’t have had to have gone through that (P5).

In addition to seeing polygenic prediction as powerful knowledge, some participants felt it would make a tangible difference in what happened next.

#### It would change our lives

Some participants were confident that polygenic scores would lead to changes for their children and themselves. Most prevalent of these was confidence that polygenic prediction would trigger early support, partly because participants believed that genetic information carries more weight than other types of evidence with decision-makers. ‘I think it will still make that support start straight away. I think as soon as you start talking about genetics people believe you, I don’t know why’ (P5).

This is interesting in light of findings which suggest that people who have experienced diagnostic genetic testing find that this does not actually make a difference to the support that they and their children receive (Lucas et al., 2022). Given that a polygenic score would identify many false positives as well as true cases, it seems even less likely that this would trigger support. If polygenic scores do become the norm, it will be important to be open with new parents about whether or not they will be used to provide access to extra support.

Some participants also felt that they would have been more sensitive parents had they known their child had an increased likelihood of being autistic. It would have encouraged them to explore alternative ways of communicating with their child and, in some cases, not pressuring them to conform to certain societal norms or expectations. ‘I think there were certain things that I can think of now through his early years that I’d have done differently had I known about autism and known about sensory issues’ (P12). Several participants talked about the judgement of others and how this information would have given them an easy way to respond and to prioritise being sensitive to the needs their child expressed.

In sum, this theme suggests that some parents of nonverbal and minimally verbal autistic children believe that polygenic prediction would increase knowledge – helping with preparation and self-education – and that it would make a difference to the child and parent’s experiences in the early years.

### Predicting autism from birth would be – at best – pointless

In contrast to the views described in Theme 3, around half of the participants believed that the prediction of autism in infancy, using polygenic scores, would make no difference to their children’s lives. For that reason, and because they saw potential for harm, they saw little value in the clinical use of polygenic scores for the likelihood of being autistic.

#### It would make little difference and could make some things worse

Participants expressed several reasons for their belief that polygenic prediction would make little difference and these included a perception that such tests would not be actionable in any meaningful way and that UK services would be unable or unwilling to respond.

It was argued that we do not have high-quality evidence-based interventions to support autistic children in their early years, and so little could be done with the information from a polygenic score. ‘It is possible that some early intervention might in the future allow autistic children to live less challenging lives but nothing like that is available at present, to my knowledge’ (P2). It is important to be clear that, in the United Kingdom, this kind of screening would never be permitted by the National Screening Committee unless it was deemed to be actionable. Participants felt that even if we had fantastic interventions that would support autistic children, they would be unlikely to receive them. This view was borne out of personal experiences of being denied access to services. P10’s frustration at this was palpable:

Yes it would’ve if it meant we would’ve had the help and support from day dot, it’s not a perfect world it doesn’t exist. Learning authorities are just a black hole; you send an email and it goes to Timbuktu, it’s just a nightmare, it really is. And if a political party came along with that as their main focus I would be absolutely campaigning on their behalf but none of them do . . .

One participant supported this view by describing how an actual diagnosis had not led to increased support – ‘even getting her diagnosis now sort of doesn’t really open any more doors’ (P19) – and so it seemed unlikely that a probability score would help.

Overall, there was a widespread view that even if participants did not object to the use of polygenic scores to predict autism in principle they did not feel there was much point in using them. Some went further and suggested that such a prediction could be psychologically harmful. The most significant harm identified was that it could spoil early experiences within families. ‘I can’t see how it would have made any difference, other than that we might not have enjoyed so completely the early months of [name]’s life’ (P2).

This feeling that there was a risk of harm that was not outweighed by clear and tangible benefits was exacerbated by a lack of trust in both science and society.

#### A lack of trust

Participants drew on their personal experiences of parenting an autistic child to conclude that they did not trust the government or society to use polygenic scores in ways that would benefit them or their children. Some also expressed distrust of the science itself.

Distrust in science is mainly related to a perceived lack of transparency in genomic research and in medical research more broadly. For instance, P17 said:

I don’t necessarily have a lot of trust in medicine, research and what have you, that they’re completely honest all of the time. And looking at history and what have you I just. . . It’s not something that I think I could be compliant in . . .

We know that concern about lack of transparency was a key issue for those who protested the launch of the Spectrum 10 K study, and there are important lessons here for researchers. First, transparency is paramount but also, the way in which transparent communication takes place needs to be designed to meet the needs of the population it addresses. Sometimes, that population – as in the case of the autistic population – will be highly heterogeneous, so multiple co-created strategies may be required. In the current data, clarity about the intended outcomes of the study was seen to be crucial. Participants recognised that providing DNA goes several steps beyond other forms of research participation, such as completing a questionnaire. As P17 put it: ‘I think DNA is a big part of somebody’. This comment makes clear that sharing DNA is dependent on a trusting relationship with science and society for some people.

Concerns went beyond science to the whole of society with some participants feeling jaded by the lack of support that they and their children had experienced to date. On the basis of their own experience of diagnosis (so a substantial step beyond prediction), P6 described how: ‘we were sort of left as two new parents who were both undergoing all of those stresses alongside being a new parent, we were trying to work out what any of this meant’.

Beyond a need for support in understanding a polygenic score and its implications there was a lack of trust that further practical support would be put in place, although several participants expressed a belief that – in a more supportive society – that could be very valuable.

If you got this test and you scored over 60% chance of becoming autistic, being nonverbal, on the spectrum so this automatically started to kick in and you had all this support there and your child was at the right place and had the right things in place for them, and the school was fully funded to be able to have a one to one with that child at all times, and you could have changes made to your house with help which is easier to get for some children that need physical changes or you could do this, this, and this then, yeah, it would be brilliant (P10).

In summary, some participants believed that polygenic scores might be useful in an ideal world but shared the belief that the world is far from ideal and, as long as that remains true, the potential for harm is greater than the potential for good. Others believed that factors such as the heterogeneity of autism, and the lack of effective interventions, would mean a polygenic score for autism would have limited utility, even in an ideal world.

### Our children’s voices are not heard in discussions about autism research

Participants expressed a view that their children’s experiences are meaningfully different to those of the most vocal members of the autistic community and that this makes them feel their children are unheard in discussions that affect them, including discussions about genomic autism research. This belief was often coupled with the idea that because their children do not have a voice – in the sense that their speech is absent or very limited – they, as parents, need to be that voice, even with the risk that they may not represent the children exactly as they would choose to be represented.

Participants used the language of severity and function level, while acknowledging that such language is unpopular within the autistic community. P6 said: ‘not everybody gets the good type of autism, the high-functioning autism’. While several participants were at pains to be clear that they did not wish to minimise the experience of others, most felt that their children faced significantly more challenges than those individuals whom they saw speaking for the autistic community on social media and elsewhere. Because they saw their children as different from the dominant autistic voice, as they perceived it, participants felt their experiences were rarely taken into account, and this exacerbated feelings of isolation. ‘It sometimes feels that the voices of high-functioning autistic people are angry and strident and do not take into account whatever their non-verbal peers may think or feel’ (P2). P15 related this specifically to the activism that took place around the launch of Spectrum 10 K:

And, so for those people that are really severely affected I think basically those people kicking off kind of robbed a portion of the autistic community of that chance, to have that research done . . . I just think it was short-sighted and selfish. That is kind of quite strong, but that is my opinion.

This illustrates the point that it is vital to acknowledge that the autistic community cannot always speak with one voice because of the enormous heterogeneity within it. This means that as researchers work to understand the needs of autistic people, they must consult with representative samples and, potentially, co-produce more than one way forward.

In advocating for their children’s experiences to be taken into account, several participants made a case that they, the parents, are the best available representatives for their children. ‘I think because he can’t express how he feels, I need to . . . be his voicepiece even more so than a child who is speaking properly’ (P4). Some acknowledged that it would be better if their child could communicate their views autonomously, but – without a means to do this – they believed that acting as their child’s voice was the next best option. ‘Yes, because I feel that it could be my view that I’m expressing more than his view but then would he have a fully developed view of it? I don’t know (P10)’. This participant was suggesting that, beyond a lack of speech, it may be difficult for a child with a learning disability to engage with a topic as complex as genomic autism research.

In summary, this theme illustrates the idea that truly inclusive autism research, including genomic autism research, has to find ways of including the ‘voices’ and experiences of autistic children and adults who are nonverbal or minimally verbal and those with associated learning disabilities. It points to the issue that developing alternative approaches to data collection, including the voices of these individuals themselves wherever possible, should be a priority for the field.

## Conclusion

We asked 20 parents of nonverbal or minimally verbal autistic children (most of whom also had significant learning difficulties) what they thought about their child participating in genomic studies of autism and about the potential risks and benefits of polygenic scores for autism. The key messages they shared were that:

- They are interested in basic science approaches to autism, including genomics, but it is important that such research focuses on developing knowledge that could help autistic people, rather than leading to eradication or cure.
- It is important for researchers to build up trust so the research has to meet the highest ethical standards, be carefully communicated and be tailored to children’s needs.
- There are mixed views on whether polygenic scores would be helpful. Some believe that they could increase knowledge and agency, and improve experiences for children and families in the earliest years. Others feel that this would only be the case if we lived in an ideal society.
- Finally, participants feel that they – and through them, their children – are not often included in discussions that affect them and that they don’t feel represented by what they perceive as the dominant autistic narrative.
